# High inter-laboratory variability in the assessment of HER2-low breast cancer: a national registry study on 50,714 Danish patients

**DOI:** 10.1186/s13058-023-01739-9

**Published:** 2023-11-09

**Authors:** Kåre Nielsen, Michael Sode, Maj-Britt Jensen, Tobias Berg, Ann Knoop, Bent Ejlertsen, Anne-Vibeke Lænkholm

**Affiliations:** 1 Department of Pathology, Zealand University Hospital, Sygehusvej 9, 4000 Roskilde, Denmark; 2 University of Copenhagen, Blegdamsvej 3B, 2100 Copenhagen, Denmark; 3 Danish Breast Cancer Group, DBCG, Rigshospitalet (Copenhagen University Hospital), Blegdamsvej 9, 2100 Copenhagen, Denmark; 4 Department of Oncology, Rigshospitalet (Copenhagen University Hospital), Blegdamsvej 9, 2100 Copenhagen, Denmark

**Keywords:** Breast cancer, Human epidermal growth factor receptor 2 (HER2), HER2 low, Variability, Reproducibility, Immunohistochemistry

## Abstract

**Background:**

Considering the recent advancements in the treatment of breast cancer with low expression of human epidermal growth factor receptor 2 (HER2), we aimed to examine inter-laboratory variability in the assessment of HER2-low breast cancer across all Danish pathology departments.

**Methods:**

From the Danish Breast Cancer Group, we obtained data on all women diagnosed with primary invasive breast cancer in 2007–2019 who were subsequently assigned for curatively intended treatment.

**Results:**

Of 50,714 patients, HER2 score and status were recorded for 48,382, among whom 59.2% belonged to the HER2-low group (score 1+ or 2+ without gene amplification), 26.8% had a HER2 score of 0, and 14.0% were HER2 positive. The proportion of HER2-low cases ranged from 46.3 to 71.8% among pathology departments (*P* < 0.0001) and from 49.3 to 65.6% over the years (*P* < 0.0001). In comparison, HER2 positivity rates ranged from 11.8 to 17.2% among departments (*P* < 0.0001) and from 12.6 to 15.7% over the years (*P* = 0.005). In the eight departments with the highest number of patients, variability in HER2-low cases increased from 2011 to 2019, although the same immunohistochemical assay was used. By multivariable logistic regression, the examining department was significantly related to both HER2 score 0 and HER2 positivity (*P* < 0.0001) but showed greater dispersion in odds ratios in the former case (range 0.25–1.41 vs. 0.84–1.27).

**Conclusions:**

Our data showed high inter-laboratory variability in the assessment of HER2-low breast cancer. The findings cast doubt on whether the current test method for HER2 is robust and reliable enough to select HER2-low patients for HER2-targeted treatment in daily clinical practice.

## Background

Until recently, therapies targeting the human epidermal growth factor receptor 2 (HER2) have been ineffective in HER2-negative breast cancer (BC) including BC with low levels of HER2 expression [1]. Recently, however, phase III results for the novel antibody–drug conjugate trastuzumab deruxtecan (T-Dxd) showed significantly improved survival in patients with metastatic HER2-low BC—defined with reference to prevailing recommendations for HER2 testing as an immunohistochemical score of 1+ or 2+ without detectable gene amplification [2, 3]. In consequence, as about 60% of primary invasive BCs belong to the HER2-low category [4–6], T-DXd may improve the outcome for a large group of patients.

The prevailing recommendations for HER2 testing from the American Society of Clinical Oncology/College of American Pathologists (ASCO/CAP) have established criteria for the immunohistochemical scores as summarized in Table 1 and supported a testing algorithm with immunohistochemistry (IHC) as the primary test and gene testing by in situ hybridization (ISH) as a supplementary test in case of score 2+ [3, 7, 8]. HER2 status is classified as positive in case of score 3+ or gene amplification and negative in case of score 0, 1+, or 2+ with normal gene status.

**Table 1:** Immunohistochemical scoring of HER2 as recommended by American Society of Clinical Oncology/College of American Pathologists (ASCO/CAP) in the guidelines first released in January 2007* and revised in November 2013† and November 2018‡

HER2 score	HER2 status	ASCO/CAP guidelines 2007*	ASCO/CAP guidelines 2013^†^	ASCO/CAP guidelines 2018^‡^
0	Negative	No staining	No staining or faint, incomplete membrane staining in ≤ 10% of tumor cells	No staining or faint, incomplete membrane staining in ≤ 10% of tumor cells
1+	Negative	Weak, incomplete membrane staining in any proportion of tumor cells	Faint, incomplete membrane staining in > 10% of tumor cells	Faint, incomplete membrane staining in > 10% of tumor cells
2+	Equivocal, dependent on supplementary in situ hybridization	Nonuniform or weak, complete, circumferential membrane staining in ≥ 10% of tumor cells or intense, complete membrane staining in ≤ 30% of tumor cells	Incomplete and/or weak to moderate, circumferential membrane staining in > 10% of tumor cells or intense, complete, circumferential membrane staining in ≤ 10% of tumor cells	Weak to moderate, complete membrane staining in > 10% of tumor cells or intense, complete membrane staining in ≤ 10% of tumor cells
3+	Positive	Uniform intense membrane staining in > 30% of tumor cells	Intense, complete, circumferential membrane staining in > 10% of tumor cells	Intense, complete, circumferential membrane staining in > 10% of tumor cells

*Wolff AC, Hammond ME, Schwartz JN, et al. American Society of Clinical Oncology/College of American Pathologists guideline recommendations for human epidermal growth factor receptor 2 testing in breast cancer. J Clin Oncol. 2007;25(1):118–145

^†^Wolff AC, Hammond ME, Hicks DG, et al. Recommendations for human epidermal growth factor receptor 2 testing in breast cancer: American Society of Clinical Oncology/College of American Pathologists clinical practice guideline update. J Clin Oncol. 2013;31(31):3997–4013

^‡^Wolff AC, Hammond MEH, Allison KH, et al. Human Epidermal Growth Factor Receptor 2 Testing in Breast Cancer: American Society of Clinical Oncology/College of American Pathologists Clinical Practice Guideline Focused Update. J Clin Oncol. 2018;36(20):2105–2122

These recommendations were, however, designed with the aim of allocating HER2-positive patients to HER2-targeted treatment with trastuzumab, and while the distinction between positive and negative cases has shown good inter-observer reproducibility [9–13], reasonable consistency among laboratories [14–16], and high concordance between biopsy and surgical specimen [17–19], the discrimination of HER2-low BC may not show similar robustness. Thus, the development of new, more effective HER2-targeted agents raises a fundamental and urgent methodological problem: Can the current test method for HER2 with reasonable reproducibility discriminate HER2-low BC? In other words, is the current test method fit to answer a different question than it was originally developed for?

To address this problem, we performed a nationwide registry study on real-world HER2 data aiming to explore inter-laboratory variability in the assessment of HER2-low BC across all Danish pathology departments.

## Methods

### Patient data

The study included women with BC diagnosed between 2007 and 2019 in Denmark.

Since 1977, the Danish Breast Cancer Group (DBCG) has hosted a nationwide clinical database on patients with primary invasive BC in Denmark, and since 2006, the database has been synchronized with the Danish register for pathology reports, Patobank, with a close-to-complete coverage of patients with histopathologically verified BC [20].

From the DBCG database, we obtained data on all female patients diagnosed between January 1, 2007, and December 31, 2019, who were subsequently assigned for curatively intended treatment according to national guidelines. Most patients with primary advanced BC were therefore not included in the study.

The following clinicopathological parameters were extracted: HER2 IHC score and, if available, HER2 gene status (reported by HER2 gene copy number and HER2/CEN17 ratio), the resulting HER2 status, age at diagnosis, histological subtype, tumor size, estrogen receptor (ER) status (reported as percentage of ER-positive tumor cells; tumor positivity defined as ≥ 1% positive tumor cells), histological grade according to the Nottingham grading system, lymph node status at time of diagnosis, and the examining pathology department. HER2 gene amplification was defined according to the ASCO/CAP recommendations in force at the time in question [3, 7, 8]. The recorded HER2 status was corrected manually in case of clear discrepancy with the recorded IHC score and gene status (*N* = 11). As we did not have access to patient files, it was not possible to retrieve missing data.

In Denmark, diagnosis and management of BC take place exclusively within the public health system, which is organized under five administrative regions: Capital Region (1.73 million inhabitants in 2013), Zealand (0.82 million), Southern Denmark (1.20 million), Central Denmark (1.27 million), and Northern Denmark (0.58 million) [21]. In consequence, all breast biopsies and surgical specimens are examined at public pathology departments, which all adhere to the national guidelines from DBCG.

In January 2007, DBCG entered recommendations on HER2-targeted treatment in the national guidelines, at first for a limited patient population and since April 2010 for all patients with HER2-positive disease [20]. Since January 2007, Danish pathologists have therefore routinely reported HER2 score and status at time of diagnosis and progression of BC. From 2005 to September 2008, the DBCG guidelines recommended a testing algorithm for HER2 which in essence was identical to the algorithm later recommended by ASCO/CAP, with reference to the HERceptin Adjuvant trial [20, 22]. The ASCO/CAP recommendations for HER2 testing released in 2007 [7] were implemented in the DBCG guidelines in September 2008, and the 2013 and 2018 revisions [3, 8] in February 2014 and December 2018, respectively [20].

In the reporting of the results, we have chosen to anonymize the pathology departments.

### IHC assays

In all Danish pathology departments, the quality of HER2 IHC and ISH is monitored semiannually as part of an external quality assurance program under the auspices of NordiQC [23]. With permission from all Danish pathology departments, we obtained data from NordiQC on assays and staining platforms used for HER2 IHC in Danish pathology departments from 2007 to 2019.

In order to include assay in a logistic regression model (see below), we entered which HER2 IHC assay every patient in the data set was assessed by, based on the departments’ semiannual reports to NordiQC. However, when the departments changed their assay, the exact date of the change was not reported. We therefore made the assumption that every change of assay was done either January 1 or July 1.

### Statistical analysis

Distribution of HER2 score and status and ER status according to region, department, and year of diagnosis was evaluated by *χ*^2^ test. Patients with unknown score/status were not included in this analysis. The proportion of patients with unknown HER2 score was evaluated separately according to department. As an alternative measure of variability, the relative difference was determined as the difference divided by the minimum value.

Multivariable logistic regression was applied to examine how department, year, and IHC assay related to HER2 score and HER2 status, respectively. We evaluated HER2 score 0 versus {1+, 2+, and 3+}, as well as HER2 positive vs. HER2 negative. Reference categories were Dept. 4 (highest patient count), year 2014 (few unknowns), and the PATHWAY assay 4B5 790-2991 (most frequently used). We ran the analysis both with all the different IHC assays and with a grouping of related assays (PATHWAY assays, HercepTest™ assays, others, and unknowns); the former gave a significantly better model and was therefore chosen. Wald *χ*^2^ statistic was used to assess the significance of the variables. We also ran the analysis with HER2 score 0 versus {1+, 2+, 3+, and score unknown} and with HER2 positive vs. {HER2 negative and status unknown}; as this only affected the estimates modestly, the results are not shown. Interactions for pair of variables were investigated in separate models.

A *P* value < 0.05 was considered statistically significant. Statistical analysis was performed using SAS Enterprise Guide 7.15, SAS Institute Inc.

## Results

From 2007 to 2019, a total of 50,714 women were diagnosed with primary invasive BC and treated with curative intent. The pathological examination was undertaken at 14 Danish pathology departments. Patient characteristics are reported in Table 2, stratified according to pathology department and administrative region. Mean age for the population was 61.2 years; 80.2% of tumors were classified as invasive ductal carcinoma; median tumor size was 16 mm; and 85.8% of tumors were ER positive. Among the three histological grades, grade II was the most frequent (42.7%). Based on sentinel node or axillary dissection, 36.3% of patients had lymph node involvement at time of diagnosis. Overall, only relatively minor differences were seen across the population.

**Table 2:** Patient characteristics stratified according to administrative region and pathology department

	Number		Age	Histological subtype					
			Mean	IDC		ILC		Other	
	*N*	%	Years	*N*	%	*N*	%	*N*	%
Capital Region	15,842	31.2	61.4	12,912	81.5	1808	11.4	1109	7.0
Dept. 1	7576	14.9	60.2	6355	83.9	835	11.0	380	5.0
Dept. 2	7982	15.7	62.4	6314	79.1	944	11.8	717	9.0
Dept. 3	284	0.6	60.9	243	85.6	29	10.2	12	4.2
Zealand	8084	15.9	61.5	6421	79.4	844	10.4	813	10.1
Dept. 4	8084	15.9	61.5	6421	79.4	844	10.4	813	10.1
Southern Denmark	11,700	23.1	61.3	9209	78.7	1204	10.3	1269	10.8
Dept. 5	3355	6.6	62.0	2459	73.3	357	10.6	532	15.9
Dept. 6	2539	5.0	62.6	2109	83.1	262	10.3	164	6.5
Dept. 7	2222	4.4	61.3	1770	79.7	221	9.9	226	10.2
Dept. 8	3584	7.1	59.7	2871	80.1	364	10.2	347	9.7
Central Denmark	10,312	20.3	60.9	8224	79.8	1101	10.7	971	9.4
Dept. 9	3709	7.3	60.2	2772	74.7	399	10.8	528	14.3
Dept. 10	743	1.5	62.3	620	83.4	63	8.5	60	8.1
Dept. 11	2027	4.0	61.0	1699	83.8	199	9.8	129	6.4
Dept. 12	3833	7.6	61.3	3133	81.8	440	11.5	254	6.6
Northern Denmark	4776	9.4	60.9	3885	81.3	513	10.7	373	7.8
Dept. 13	3097	6.1	60.6	2483	80.2	352	11.4	259	8.4
Dept. 14	1679	3.3	61.5	1402	83.5	161	9.6	114	6.8
In total	50,714	100	61.2	40,651	80.2	5470	10.8	4535	8.9

	Size	Estrogen receptor positivity					
	Median	0%		1–9%		> 9%	
	mm	*N*	%	*N*	%	*N*	%
Capital Region	16	2195	13.9	226	1.4	13,382	84.4
Dept. 1	16	1103	14.6	152	2.0	6300	83.1
Dept. 2	16	1051	13.2	68	0.9	6846	85.7
Dept. 3	20	41	14.4	6	2.1	236	83.1
Zealand	15	1094	13.5	134	1.7	6831	84.4
Dept. 4	15	1094	13.5	134	1.7	6831	84.4
Southern Denmark	16	1664	14.2	198	1.7	9815	83.8
Dept. 5	16	459	13.7	54	1.6	2833	84.4
Dept. 6	16	351	13.8	33	1.3	2152	84.7
Dept. 7	17	287	12.9	52	2.3	1877	84.3
Dept. 8	15	567	15.8	59	1.6	2953	82.4
Central Denmark	16	1375	13.3	159	1.5	8756	84.8
Dept. 9	15	432	11.6	62	1.7	3199	85.9
Dept. 10	18	135	18.2	6	0.8	602	81.0
Dept. 11	17	237	11.7	39	1.9	1748	86.2
Dept. 12	15	571	14.9	52	1.4	3207	83.6
Northern Denmark	16	690	14.4	81	1.7	3996	83.1
Dept. 13	16	433	14.0	54	1.7	2607	83.4
Dept. 14	18	257	15.3	27	1.6	1389	82.5
In total	16	7018	13.8	798	1.6	42,780	84.2

	Histological grade						LN metastasis	
	I		II		III			
	*N*	%	*N*	%	*N*	%	*N*	%
Capital Region	4394	27.7	6743	42.6	3328	21.0	5767	36.4
Dept. 1	1812	23.9	3423	45.2	1815	24.0	2785	36.8
Dept. 2	2511	31.5	3201	40.1	1433	18.0	2813	35.2
Dept. 3	71	25.0	119	41.9	80	28.2	169	59.5
Zealand	1764	21.8	3577	44.2	1784	22.1	2655	32.8
Dept. 4	1764	21.8	3577	44.2	1784	22.1	2655	32.8
Southern Denmark	3115	26.6	4871	41.6	2314	19.8	4104	35.1
Dept. 5	933	27.8	1165	34.7	685	20.4	1197	35.7
Dept. 6	700	27.6	1024	40.3	630	24.8	934	36.8
Dept. 7	581	26.1	1020	45.9	366	16.5	755	34.0
Dept. 8	901	25.1	1662	46.4	633	17.7	1218	34.0
Central Denmark	2640	25.6	4502	43.7	2052	19.9	3943	40.1
Dept. 9	808	21.8	1547	41.7	710	19.1	1387	37.4
Dept. 10	213	28.7	287	38.6	182	24.5	354	47.6
Dept. 11	546	26.9	923	45.5	423	20.9	753	37.1
Dept. 12	1073	28.0	1745	45.5	737	19.2	1449	37.8
Northern Denmark	1109	23.2	1963	41.1	1267	26.5	1921	40.2
Dept. 13	805	26.0	1175	37.9	812	26.2	1210	39.1
Dept. 14	304	18.1	788	46.9	455	27.1	711	42.3
In total	13,022	25.7	21,656	42.7	10,745	21.2	18,390	36.3

*LN* lymph node, *IDC* invasive ductal carcinoma, *ILC* invasive lobular carcinoma

**Fig. 1:**
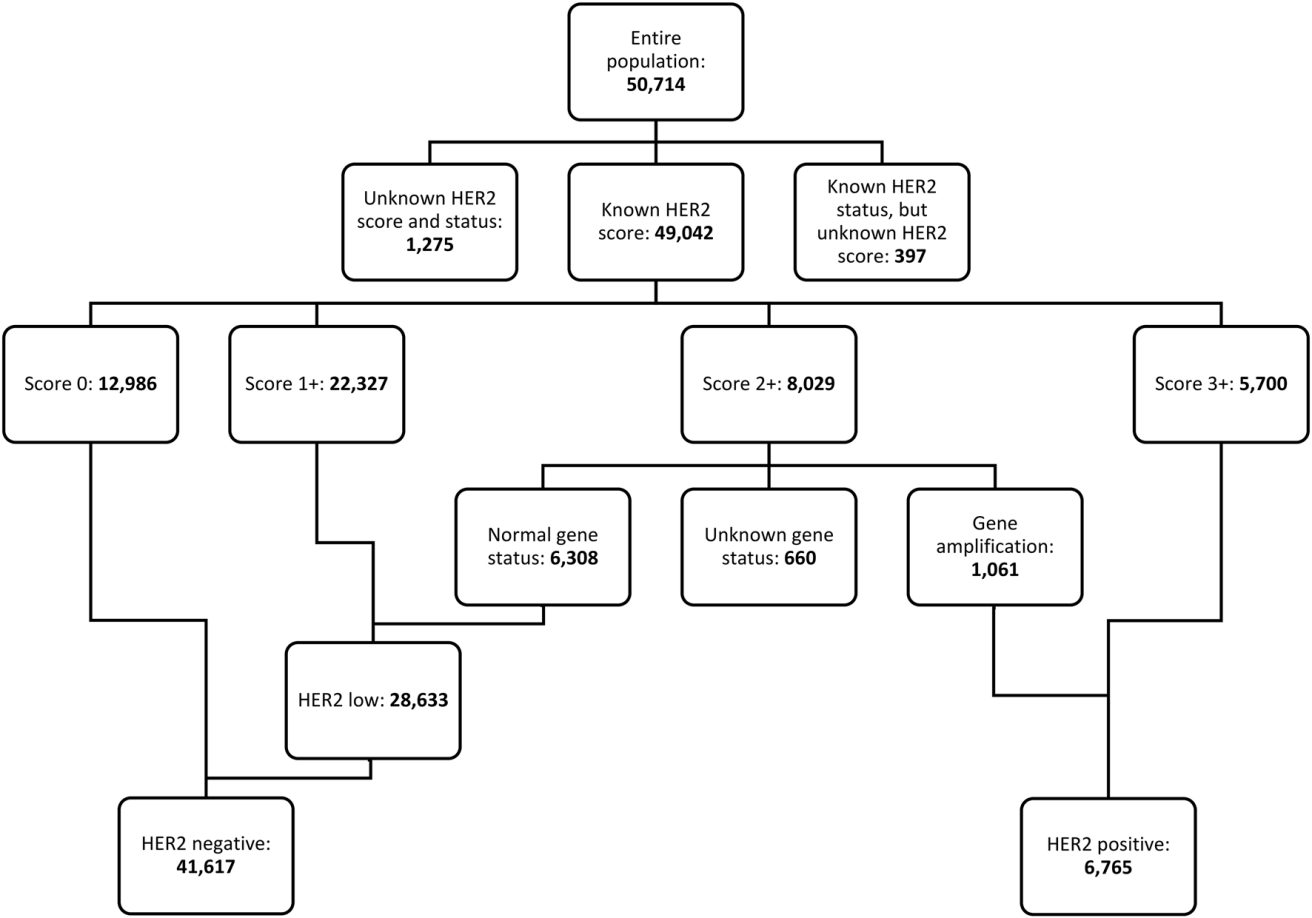
Block diagram of the population. Two patients with a score of 0 and two with a score of 1 + were classified as HER2 positive due to gene amplification; these four patients are not plotted explicitly in the chart

For 49,042 patients (96.7%), HER2 IHC score was recorded in the DBCG database, and for 48,382 patients (95.4%), both HER2 score and HER2 status were recorded, as schematized in Fig. 1. For 397 patients (0.78%), HER2 status, but not HER2 score, was recorded; among these, 303 (76.3%) were negative of HER2 and 94 (23.7%) positive, and 371 (94.9%) had a recording of HER2/CEN17 ratio. Among the 8029 patients with a score of 2+, 6308 (78.6%) had normal gene status, 1061 (13.2%) had gene amplification, and 660 (8.2%) had unknown gene status. Among patients with a score of 0 or 1+, gene status was reported for 281, among whom 277 had normal gene status and four had gene amplification (two with a score of 0 and two with a score of 1+, hence classified as HER2 positive).

### Distribution of HER2 scores

Table 3 shows how HER2 was scored in Danish pathology departments from 2007 to 2019. The distribution of the scores varied significantly among regions, departments, and years (*P* < 0.0001 in all cases). When patients with unknown HER2 score were left out of account, the relative frequency of the scores ranged among departments from 10.7 to 38.1% for score 0, from 35.8 to 58.8% for score 1+, from 6.7 to 31.0% for score 2+, and from 9.5 to 15.6% for score 3+. The inter-laboratory variability for scores 0 and 2+ corresponded to a very high relative difference of 2.6 and 3.6, respectively. Inter-annually, frequencies ranged from 20.1 to 33.7% for score 0, from 40.4 to 51.3% for score 1+, from 12.9 to 18.8% for score 2+, and from 10.8 to 13.2% for score 3+. Surprisingly, the adjusted definition of score 0 in the 2013 revision of the ASCO/CAP guidelines (cf. Table 1) did not increase the frequency of score 0 (26.6% in the years 2007–2013 vs. 26.4% in the years 2014–2019).

**Table 3:** Distribution of HER2 scores across administrative regions and pathology departments

HER2 score	0		1+		2+		3+		NA		In total
	*N*	% (excl. NA)	*N*	% (excl. NA)	*N*	% (excl. NA)	*N*	% (excl. NA)	*N*	%	*N*
Capital Region	4018	25.4 (26.0)	6834	43.1 (44.2)	2739	17.3 (17.7)	1858	11.7 (12.0)	393	2.5	15,842
Dept. 1	1996	26.3 (26.6)	3286	43.4 (43.8)	1316	17.4 (17.5)	909	12.0 (12.1)	69	0.9	7576
Dept. 2	1944	24.4 (25.2)	3464	43.4 (44.9)	1384	17.3 (17.9)	921	11.5 (11.9)	269	3.4	7982
Dept. 3	78	27.5 (34.1)	84	29.6 (36.7)	39	13.7 (17.0)	28	9.9 (12.2)	55	19.4	284
Zealand	2497	30.9 (31.5)	3341	41.3 (42.1)	1182	14.6 (14.9)	911	11.3 (11.5)	153	1.9	8084
Dept. 4	2497	30.9 (31.5)	3341	41.3 (42.1)	1182	14.6 (14.9)	911	11.3 (11.5)	153	1.9	8084
Southern Denmark	2880	24.6 (24.8)	6250	53.4 (53.8)	1206	10.3 (10.4)	1289	11.0 (11.1)	75	0.6	11,700
Dept. 5	1128	33.6 (33.9)	1523	45.4 (45.8)	331	9.9 (10.0)	344	10.3 (10.3)	29	0.9	3355
Dept. 6	653	25.7 (25.8)	1430	56.3 (56.5)	169	6.7 (6.7)	277	10.9 (11.0)	10	0.4	2539
Dept. 7	337	15.2 (15.3)	1295	58.3 (58.8)	343	15.4 (15.6)	229	10.3 (10.4)	18	0.8	2222
Dept. 8	762	21.3 (21.4)	2002	55.9 (56.1)	363	10.1 (10.2)	439	12.2 (12.3)	18	0.5	3584
Central Denmark	1988	19.3 (20.4)	4235	41.1 (43.4)	2427	23.5 (24.9)	1105	10.7 (11.3)	557	5.4	10,312
Dept. 9	621	16.7 (17.9)	1554	41.9 (44.7)	970	26.2 (27.9)	330	8.9 (9.5)	234	6.3	3709
Dept. 10	101	13.6 (21.7)	243	32.7 (52.3)	50	6.7 (10.8)	71	9.6 (15.3)	278	37.4	743
Dept. 11	215	10.6 (10.7)	886	43.7 (44.0)	625	30.8 (31.0)	287	14.2 (14.3)	14	0.7	2027
Dept. 12	1051	27.4 (27.6)	1552	40.5 (40.8)	782	20.4 (20.6)	417	10.9 (11.0)	31	0.8	3833
Northern Denmark	1603	33.6 (37.4)	1667	34.9 (38.9)	475	9.9 (11.1)	537	11.2 (12.5)	494	10.3	4776
Dept. 13	1126	36.4 (38.1)	1191	38.5 (40.3)	306	9.9 (10.4)	329	10.6 (11.1)	145	4.7	3097
Dept. 14	477	28.4 (35.9)	476	28.4 (35.8)	169	10.1 (12.7)	208	12.4 (15.6)	349	20.8	1679
In total	12,986	25.6 (26.5)	22,327	44.0 (45.5)	8029	15.8 (16.4)	5700	11.2 (11.6)	1672	3.3	50,714

*NA* not available

**Fig. 2:**
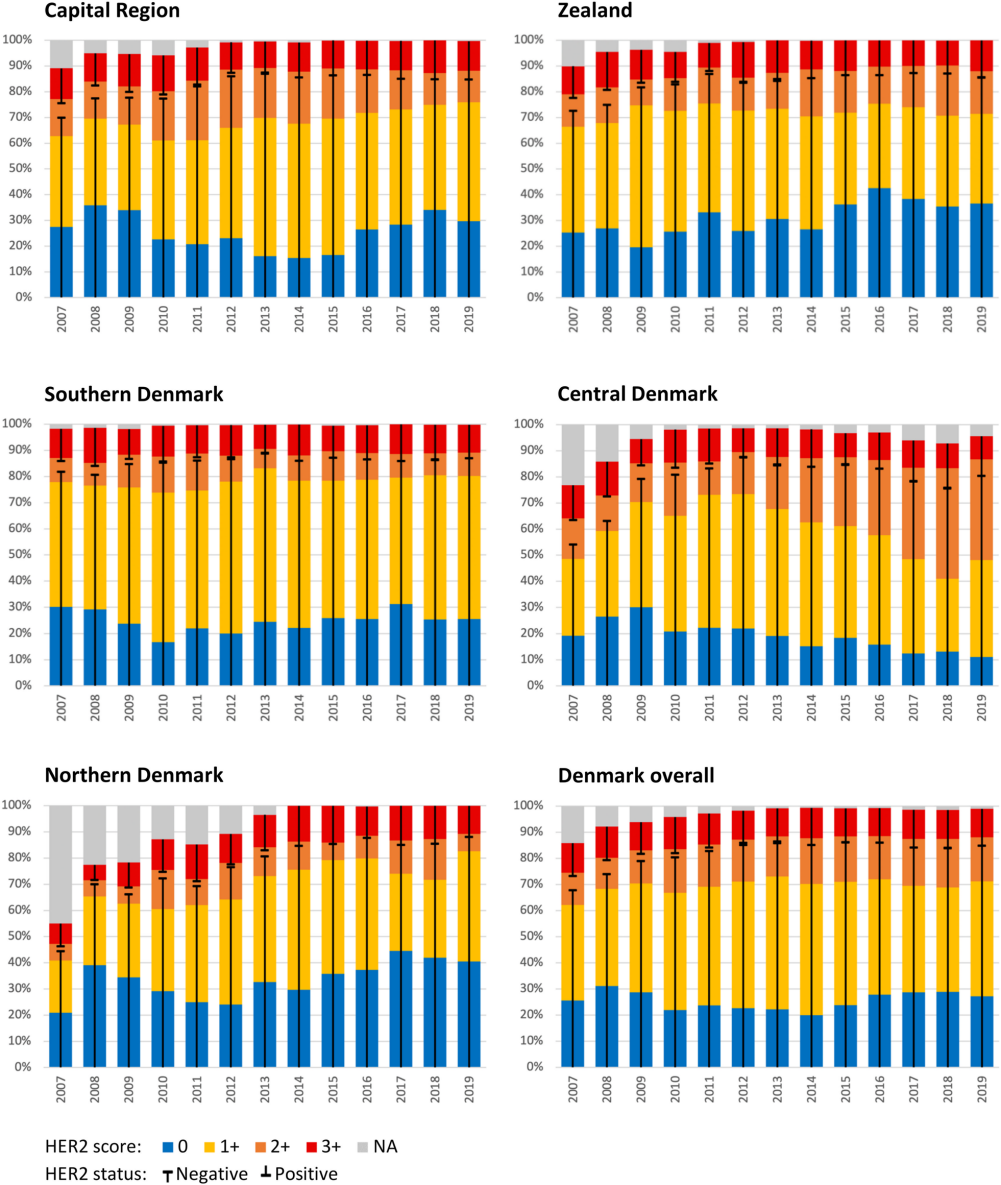
Distribution of HER2 scores over time in the five administrative regions of Denmark. (NA, not available)

In Fig. 2, the distribution of the scores is illustrated over time for the five administrative regions. Striking differences and trends appear: Thus, from 2011 onwards, the frequency of score 2+ increased in Central Denmark and declined in Capital Region, and from 2009 onwards, the frequency of score 0 declined in Central Denmark. Likewise, for the individual departments, different trends were seen across the years; e.g., in the years 2017–2019, the frequency of score 0 increased from 8.9 to 12.8% to 28.6% in Dept. 11 and decreased from 56.5 to 46.2 to 40.4% in Dept. 13 (data not shown).

In addition to this, the proportion of patients with unknown HER2 score differed significantly among departments (*P* < 0.0001). Here, three departments stood out: Dept. 3, 10, and 14 with 19.4% (*N* = 55), 37.4% (*N* = 278), and 20.8% (*N* = 349) unknowns, respectively, as compared to 2.1% at the other 11 departments. Overall, in the entire population, the number of patients with unknown HER2 score declined from 2007 to 2013—and from 2012 onwards, the proportion was < 1% every year. In this context, Dept. 9 deviated from the overall picture, as the proportion increased in the last part of the study period, from 2.5% in the years 2007–2014 (*N* = 57) to 12.1% in the years 2015–2019 (*N* = 234). Of the 397 patients with recorded HER2 status but unknown HER2 score, 200 came from Dept. 9 (all with recorded HER2/CEN17 ratio and 171 from the years 2015–2019) and 130 from Dept. 2 (109 with recorded ratio).

### Variability in HER2 status and HER2-low BC

Table 4 shows variability in HER2 status for the 48,382 patients with recordings of both HER2 score and HER2 status. Among these patients, 6765 (14.0%) had positive HER2 status and 28,633 (59.2%) belonged to the HER2-low group. HER2 positivity rates ranged from 13.1 to 14.6% among regions (*P* = 0.004, relative difference 0.11), from 11.8 to 17.2% among departments (*P* < 0.0001, relative difference 0.46), and from 12.6 to 15.7% over the years (*P* = 0.005, relative difference 0.25).

**Table 4:** Variability in HER2 status among patients with recordings of both HER2 score and HER2 status

HER2 status	Negative						Positive		
	*Score 0*		*HER2 low*		*In total*				
	*N*	%	*N*	%	*N*	%	*N*	%	*N*
Capital Region	4017	26.3	9038	59.2	13,055	85.6	2202	14.4	15,257
Dept. 1	1996	26.7	4394	58.8	6390	85.5	1087	14.5	7477
Dept. 2	1943*	25.7	4532	60.0	6475	85.7	1083	14.3	7558
Dept. 3	78	35.1	112	50.5	190	85.6	32	14.4	222
Zealand	2497	31.9	4261	54.4	6758	86.2	1079	13.8	7837
Dept. 4	2497	31.9	4261	54.4	6758	86.2	1079	13.8	7837
Southern Denmark	2879	25.0	7134	61.9	10,013	86.9	1503	13.1	11,516
Dept. 5	1128	34.3	1749	53.2	2877	87.6	408	12.4	3285
Dept. 6	652*	26.1	1537	61.6	2189	87.8	305	12.2	2494
Dept. 7	337	15.5	1559	71.6	1896	87.1	282	12.9	2178
Dept. 8	762	21.4	2289	64.3	3051	85.7	508	14.3	3559
Central Denmark	1988	20.8	6155	64.5	8143	85.4	1397	14.6	9540
Dept. 9	621	18.2	2389	70.0	3010	88.2	404	11.8	3414
Dept. 10	101	22.9	265	60.1	366	83.0	75	17.0	441
Dept. 11	215	11.0	1404	71.8	1619	82.8	336	17.2	1955
Dept. 12	1051	28.2	2097	56.2	3148	84.4	582	15.6	3730
Northern Denmark	1603	37.9	2045	48.3	3648	86.2	584	13.8	4232
Dept. 13	1126	38.4	1442	49.2	2568	87.6	363	12.4	2931
Dept. 14	477	36.7	603	46.3	1080	83.0	221	17.0	1301
In total	12,984	26.8	28,633	59.2	41,617	86.0	6765	14.0	48,382

*Discrepancy from Table 3 due to patients with a score of 0 and concurrent gene amplification, thus classified as HER2 positive

**Fig. 3:**
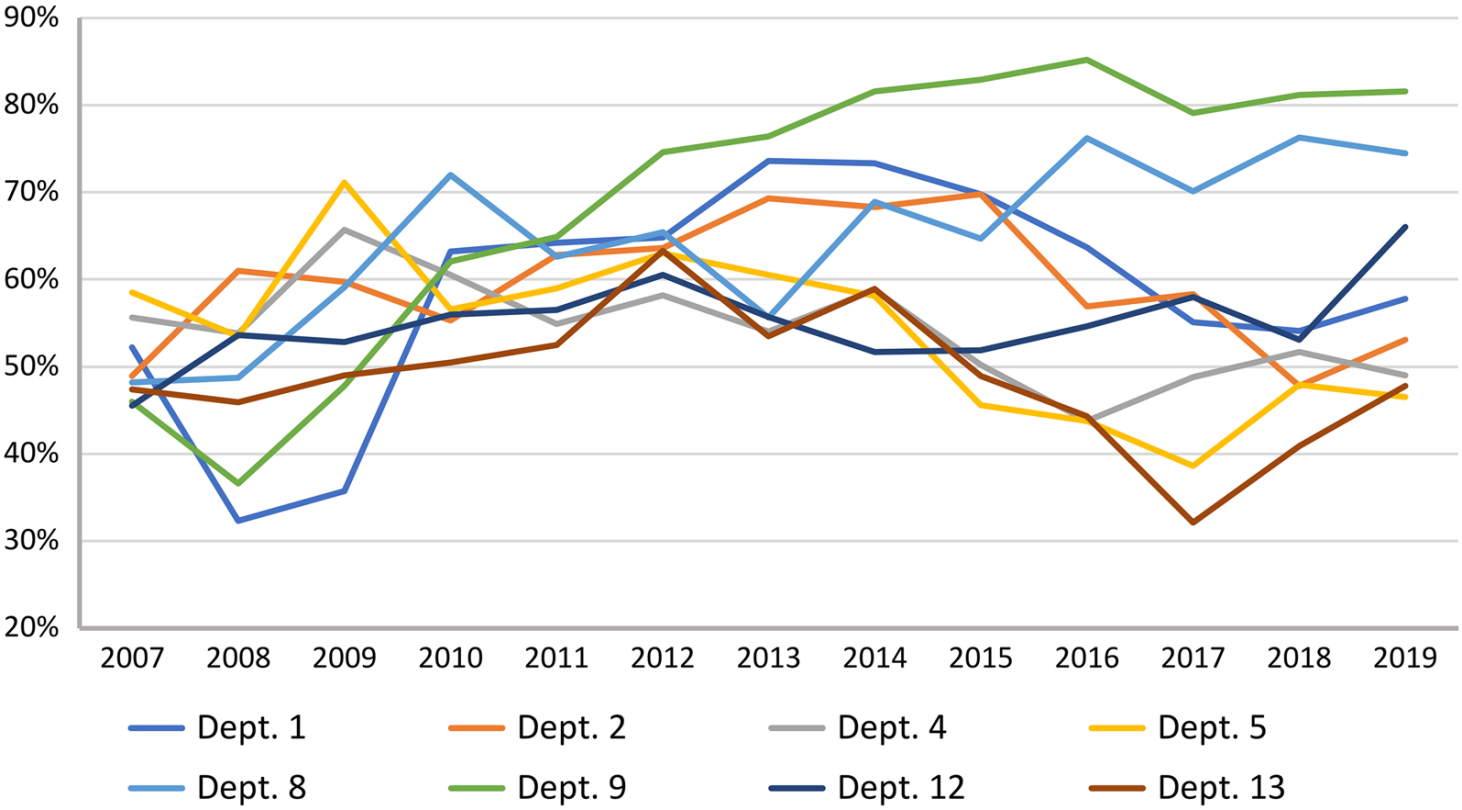
Frequency of HER2-low breast cancer among patients with recordings of both HER2 score and status in the eight pathology departments with more than 3000 breast cancer patients

The proportion of HER2-low cases ranged from 48.3 to 64.5% among regions (*P* < 0.0001, relative difference 0.34), from 46.3 to 71.8% among departments (*P* < 0.0001, relative difference 0.55), and from 49.3 to 65.6% over the years (*P* < 0.0001, relative difference 0.33). When the eight pathology departments with more than 3000 BC patients were considered separately, the frequency of score 0 ranged from 18.1 to 38.4% and the proportion of HER2-low cases from 49.2 to 70.0% (*P* < 0.0001 in both cases). In Fig. 3, the HER2-low rates in these eight departments are illustrated over time. As it appears, the dispersion increased from 2011 to 2019: In 2011, the range was 52.5–64.9%, while in 2019, it was 46.5–81.6%. In the three departments with the highest patient count (Dept. 1, 2, and 4), HER2-low rates ranged from 54.4 to 60.0% (*P* < 0.0001).

Of note, HER2 positivity rates showed only slightly higher variability than ER positivity rates (cf. Table 2), which ranged from 85.4 to 86.5% among regions (*P* = 0.22), from 81.8 to 88.2% among departments (*P* < 0.0001), and from 82.0 to 87.4% over the years (*P* < 0.0001).

### IHC assays

**Fig. 4:**
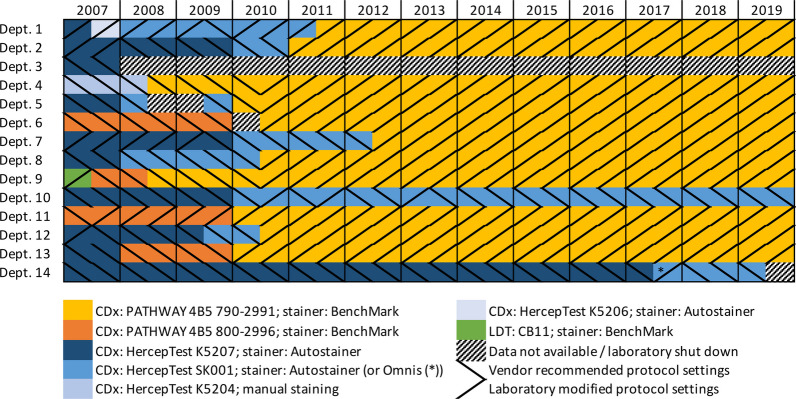
Assays and staining platforms for HER2 immunohistochemistry (data kindly provided by NordiQC). (CDx, Companion diagnostics; LDT, Laboratory developed test)

Figure 4 shows the assays and staining platforms used for HER2 IHC in all Danish pathology laboratories. A general movement from HercepTest™ antibodies K5207 and SK001 (Dako/Agilent) toward PATHWAY antibody 4B5 790-2991 (Ventana/Roche) is noticed. Indeed, in 2007, 11 out of 14 laboratories used different HercepTest™ assays, while from 2012 onwards, 11 out of 13 laboratories used 4B5 790-2991, including the eight departments with the highest number of BC patients.

### Multivariable logistic regression

By multivariable logistic regression, we examined the impact of department, year, and IHC assay on the odds of being classified as HER2 positive or HER2 score 0, respectively, as reported in Table 5. Besides an analysis of the entire study period (2007–2019), we did an analysis of the last six years alone (2014–2019), as this period gave a more present picture and only covered two guideline editions with very similar scoring criteria (cf. Table 1). In the analysis of the last six years, we excluded Dept. 3 due to shutdown of the laboratory in January 2012 and Dept. 10 due to a patient count of only 22.

**Table 5:** Multivariable logistic regression testing the impact of department, year, and immunohistochemical assay on the odds of being classified as either HER2 positive or HER2 score 0—performed for both the entire study period and for the last six years

	2007–2019				2014–2019			
	Positive versus negative		0 versus 1+, 2+, and 3+		Positive versus negative		0 versus 1+, 2+, and 3+	
	OR	95% CL	OR	95% CL	OR	95% CL	OR	95% CL
Dept. 1	1.06	0.96–1.17	0.71	0.66–0.77	1.13	0.99–1.28	0.52	0.47–0.57
Dept. 2	1.04	0.94–1.15	0.74	0.68–0.80	1.00	0.88–1.14	0.68	0.61–0.75
Dept. 3	0.91	0.61–1.36	0.97	0.72–1.31	–*		–*	
Dept. 4	1		1		1		1	
Dept. 5	0.88	0.78–1.00	1.12	1.03–1.23	0.85	0.72–1.01	1.26	1.12–1.42
Dept. 6	0.84	0.73–0.97	0.77	0.69–0.86	0.91	0.75–1.12	0.76	0.66–0.88
Dept. 7	0.89	0.77–1.03	0.38	0.33–0.43	1.01	0.82–1.23	0.30	0.25–0.36
Dept. 8	1.04	0.92–1.17	0.52	0.47–0.57	1.15	0.97–1.36	0.26	0.22–0.31
Dept. 9	0.87	0.77–0.98	0.46	0.42–0.51	0.98	0.83–1.16	0.11	0.09–0.14
Dept. 10	1.20	0.91–1.58	0.57	0.45–0.72	–*		–*	
Dept. 11	1.27	1.10–1.46	0.25	0.22–0.30	0.98	0.79–1.23	0.19	0.15–0.24
Dept. 12	1.16	1.03–1.30	0.83	0.76–0.91	1.45	1.25–1.68	0.59	0.52–0.66
Dept. 13	0.86	0.76–0.98	1.36	1.24–1.49	0.91	0.75–1.09	1.27	1.12–1.44
Dept. 14	1.08	0.87–1.34	1.41	1.19–1.67	1.22	0.86–1.73	0.80	0.61–1.06
Year 2007	0.91	0.74–1.14	2.39	2.01–2.83	–		–	
Year 2008	0.96	0.81–1.14	2.42	2.11–2.77	–		–	
Year 2009	0.87	0.74–1.01	1.92	1.70–2.17	–		–	
Year 2010	1.03	0.90–1.19	1.11	0.99–1.25	–		–	
Year 2011	0.96	0.84–1.09	1.27	1.13–1.42	–		–	
Year 2012	0.87	0.77–1.00	1.19	1.06–1.33	–		–	
Year 2013	0.89	0.78–1.02	1.15	1.03–1.29	–		–	
Year 2014	1		1		1		1	
Year 2015	0.91	0.80–1.04	1.25	1.12–1.40	0.91	0.80–1.04	1.25	1.12–1.40
Year 2016	0.93	0.82–1.06	1.55	1.39–1.73	0.94	0.82–1.07	1.54	1.38–1.72
Year 2017	1.04	0.91–1.18	1.61	1.45–1.79	1.04	0.92–1.19	1.64	1.47–1.82
Year 2018	1.06	0.94–1.20	1.60	1.44–1.78	1.06	0.93–1.21	1.62	1.45–1.80
Year 2019	1.03	0.90–1.17	1.46	1.31–1.62	1.01	0.89–1.15	1.45	1.30–1.62
Assay 4B5 790–2991	1		1		1		1	
Assay 4B5 800–2996	1.17	0.97–1.43	0.81	0.70–0.94	–		–	
Assay CB11	2.25	1.17–4.33	0.62	0.32–1.18	–		–	
Assay K5204	1.15	0.88–1.50	0.57	0.47–0.70	–		–	
Assay K5206	1.31	0.88–1.94	0.78	0.56–1.07	–		–	
Assay K5207	1.24	1.05–1.48	0.74	0.65–0.85	1.13	0.74–1.71	1.04	0.74–1.45
Assay SK001	1.00	0.88–1.14	1.27	1.15–1.41	^†^		^†^	
Unknown assay	1.09	0.77–1.54	0.44	0.33–0.59	–		–	

*OR* odds ratio, *CL* confidence level

*Excluded from the model due to shutdown of the laboratory (Dept. 3) or low patient count (Dept. 10)

^†^As SK001 was only used by Dept. 14, the estimates for SK001 are identical to the estimates for Dept. 14

The examining pathology department was significantly related to HER2 positivity (*P* < 0.0001 for both 2007–2019 and 2014–2019) with odds ratios (ORs) ranging from 0.84 (95% confidence level (CL) 0.73–0.97) to 1.27 (95% CL 1.10–1.46) among all departments and from 0.86 (95% CL 0.76–0.98) to 1.16 (95% CL 1.03–1.30) for the eight departments with the highest patient count. Similarly, the examining pathology department had a significant impact on odds for score 0 (*P* < 0.0001 for both 2007–2019 and 2014–2019) with ORs ranging from 0.25 (95% CL 0.22–0.30) to 1.41 (95% CL 1.19–1.67) among all departments and from 0.46 (95% CL 0.42–0.51) to 1.36 (95% CL 1.24–1.49) for the eight departments with the highest patient count.

In the analysis of the entire study period, IHC assay was significantly related to HER2 score 0 (*P* < 0.0001) but not HER2 positivity (*P* = 0.08), whereas the assay had no significant impact in the period 2014–2019, where 11 out of 12 laboratories in the model used the same assay (*P* > 0.5 for both HER2 positivity and score 0). Year of diagnosis was significantly related to HER2 score 0, both in the entire period and in the last six years (*P* < 0.0001 in both cases), but only to HER2 positivity in the analysis of the entire period (*P* = 0.01 vs. *P* = 0.15 for the last six years).

Tests for interactions in the model of HER2 positive vs. HER2 negative showed significant interactions in the years 2007–2019 between department and year (*P* = 0.002, indicating that HER2 positivity rates developed differently at the departments across the years) and department and assay (*P* < 0.001, indicating that the impact of assay on HER2 positivity differed among departments) but not between assay and year (*P* = 0.33, indicating that the impact of assay was stable across the years); for the years 2014–2019, no significant interactions were found. In the model of score 0 versus 1+, 2+, and 3+, significant interactions were found between department, year, and assay for the years 2007–2019 (*P* < 0.0001 for both department/year, department/assay, and assay/year, indicating that the frequency of score 0 developed differently at the departments across the years, as exemplified above, and that the impact of assay differed among departments and across the years); for the years 2014–2019, significant interactions were demonstrated between department and year (*P* < 0.0001) and assay and year (*P* = 0.002).

## Discussion

With the development of new, more effective anti-HER2 agents, patients with HER2-low BC may now benefit from HER2-targeted treatment. These advances, however, call into question whether the current test method for HER2 with reasonable reproducibility can discriminate HER2-low disease [24].

We performed a nationwide registry study on 50,714 women diagnosed with BC in the period 2007–2019, using data from daily clinical practice across all Danish pathology departments. HER2 score and status were recorded for 48,382 patients (95.4%), among whom 59.2% belonged to the HER2-low group and 14.0% were positive of HER2. The proportion of patients with HER2-low disease varied by 25.5 percentage points among departments (range 46.3–71.8%, relative difference 0.55) and 16.3 percentage points over the years (range 49.3–65.6%, relative difference 0.33). Notably, in the eight pathology departments with the highest number of patients, variability in HER2-low cases increased from 2011 onwards, although the same IHC assay and staining platform were used. In comparison, the proportion of HER2-positive cases varied by 5.4 percentage points among departments (range 11.8–17.2%, relative difference 0.46) and 3.1 percentage points over the years (range 12.6–15.7%, relative difference 0.25). By multivariable logistic regression, the examining pathology department was significantly related to both HER2 score 0 and HER2 positivity (*P* < 0.0001) but showed greater dispersion in ORs in the former case (range 0.25–1.41 vs. 0.84–1.27 among all departments). Overall, IHC assay and year of diagnosis were stronger predictors of HER2 score 0 than of HER2 positivity.

Consequently, the assessment of HER2-low BC showed markedly higher inter-laboratory variability than the assessment of HER2-positive disease, although the relative differences were equally high. The findings cast doubt on whether the current test method can be used for allocating patients with HER2-low BC to HER2-targeted treatment in daily clinical practice. With the ambition of targeting HER2-low BC therapeutically, reliable and robust delimitation of score 1+ from score 0 is essential as false results may lead to misassignment for treatment or no treatment. Therefore, if reproducibility is not improved significantly, our data may support that T-DXd is offered to all patients with metastatic HER2-negative BC, rather than to HER2-low patients alone, given the high efficacy of T-DXd reported by Modi et al. [2]. Indeed, phase II results for T-DXd did show some activity in BC with a score of 0 [25], supposedly primarily in cases with sporadic (≤ 10%) incomplete membrane reaction; this subgroup is therefore also eligible for randomization in the ongoing phase III trial for T-DXd, DESTINY-Breast06 (ClinicalTrials.gov ID: NCT04494425). Our findings stress the need for standardized procedures, as well as further investigation of assay interchangeability. In addition, our findings support the reassessment of previously stained HER2 slides if a metastatic lesion cannot be biopsied. The overall proportion of HER2-low cases in our study is in line with other population-based investigations [5, 6].

Limitations to the study include variability in cases of unreported HER2 score among departments. This could be a source of bias, as it is not a given that these patients showed similar patterns of HER2 expression as patients with known HER2 score. In fact, the group of patients with unreported HER2 score but recorded HER2 gene status (*N* = 371) was enriched of HER2-positive cases (23.7%). Most of these patients came from Dept. 9 (*N* = 200) and 2 (*N* = 109), suggesting some local underreporting of score 2+/3+. However, the overall data completeness was high and improved during the years.

The high variability in HER2-low BC presented in the current study is consistent with recent data from CAP's quality assurance program, where tissue microarray cores from 80 BC cases were stained and scored for HER2 at 1400 laboratories [26]. Here, 15 out of 56 cases considered as score 0 or 1+ had less than 70% inter-rater agreement. In the same study, a data set of 170 scanned slides assessed by 18 experienced pathologists showed only 26% concordance between HER2 score 0 and 1+ as compared to 58% between score 2+ and 3+ [26].

This and other studies suggest that the variability demonstrated in the present study is in large part attributable to variability in the evaluation of the IHC stains [26–30]. Indeed, the scoring methodology is a matter of subjective interpretation, and the scores have in several studies shown considerable inter-rater variability, especially (as common logic would dictate) the intermediate scores [28, 29, 31–34]. As regards HER2-low disease, the decisive distinction goes between score 0 and 1+, which has until now been clinically inconsequential; accordingly, for this distinction, pathologists may have adhered less rigorously to the ASCO/CAP criteria and may only rarely have conferred cases with colleagues. This may have increased variability in HER2-low rates further. In light of this, ASCO/CAP now recommends that cases close to the interpretive threshold between score 0 and 1+ be assessed by two pathologists at 40× magnification [35].

In addition to this, discrepancies in staining protocols and assays—i.e., analytical differences—and in the handling of the tissue—pre-analytical differences—may have contributed to the high variability. Thus, the currently available IHC assays are designed for detecting HER2-positive cases, where the number of HER2 receptors per cell is 25–100 times higher than in normal breast tissue and in BC cases with a score of 0 or 1+ [36, 37]. It is therefore not surprising that the assays lack both sensitivity and specificity for capturing the low-HER2 dynamic range. Moreover, different IHC assays show different staining patterns, as recently demonstrated by Agilent Technologies whose latest HercepTest™ assay reportedly lowered the frequency of score 0 by 37.5% [38]. However, from 2012 to 2019, 11 out of 13 Danish pathology departments used the same assay and staining platform for HER2 IHC (cf. Fig. 4), although possibly with some discrepancies in protocols, and in all Danish pathology departments, the quality of HER2 IHC and ISH is subject to close external control in a common quality assurance program [23]. For pre-analytical factors such as time to ischemia, fixation, tissue preparation, section thickness, choice of control tissue, and whole-slide vs. tissue microarray evaluation, international standards are widely implemented, yet the significance of these factors is only sporadically monitored. Among these, cold ischemia and underfixation are probably the best elucidated in BC, as delayed and poor fixation reduced HER2 immunoreaction in several studies [39–41]. In fact, fixation itself is reported to reduce HER2 receptor antigenicity [42, 43]. The impact of these factors may be relatively greater in the low-HER2 range.

Regarding possible solutions to the high variability in HER2-low rates, we consider it plausible that, in itself, increased awareness and a formal redefinition of the dichotomous HER2 status (as recently proposed by European Society for Medical Oncology [44]) will help reduce differences in scoring practice [24]. Moreover, training of pathologists, possibly assisted by digital learning tools, could improve concordance, just as digital image analysis calibrated to distinguish score 0 from 1+ could be a helpful supplement to light microscopy [32, 45, 46]. In addition to this, our data may indicate that central review could be part of the solution, as the three departments with the highest patient count only differed by 5.6 percentage points in HER2-low rates; this comes, however, at a cost in terms of turnaround time. Finally, the introduction of novel molecular analyses must be considered, e.g., as an add-on in case of score 0 or 1+. In theory, quantitative measurements of the treatment target, i.e., the HER2 receptor, or a closely related surrogate marker such as mRNA would be preferable to biomarkers reflecting more upstream molecular events, e.g., gene amplification. Kennedy et al. measured the number of HER2 receptors in HER2-negative tumors by means of targeted mass spectrometry and showed an implied positive correlation with IHC score, although with great variance around the trend and great overlap between the scores (thereby possibly illustrating the inaccuracy of the current test method in the low end of the scoring system) [47]. Recently, Moutafi et al [48] introduced quantitative immunofluorescence of HER2 in HER2-low BC showing good association with targeted mass spectrometry and decent association with IHC. RNA-based methods remain to be investigated properly in HER2-low disease [47, 49] but have previously shown conflicting results in gene amplified BC [50]. In contrast to IHC, proteomic and transcriptomic methods for HER2 quantification provide a normal range for HER2 expression. Our findings highlight the need for further investigation into these methods in search of a quantitative, clinically feasible, and reproducible alternative to IHC.

## Conclusions

The findings of this nationwide real-world data study showed high inter-laboratory variability in the assessment of HER2-low BC. The results cast doubt on whether the current test method for HER2 is robust and reliable enough to select HER2-low patients for HER2-directed treatment in daily clinical practice. Our data stress the need for standardized procedures, as well as further research into new, quantitative methods for HER2-low testing.

## Data Availability

The data analyzed in this study are administered by The Danish Clinical Quality Program (https://rkkp.dk) and can be acquired by applying this program. Restrictions apply to the availability of the data as the data are regarded as personally identifiable information.

## Abbreviations

ASCO: American Society of Clinical Oncology
BC: Breast cancer
CAP: College of American Pathologists
CL: Confidence level
DBCG: Danish Breast Cancer Group
ER: Estrogen receptor
HER2: Human epidermal growth factor receptor 2
IHC: Immunohistochemistry
ISH: In situ hybridization
OR: Odds ratio
T-Dxd: Trastuzumab deruxtecan
